# Efficacy of LL-37 cream in enhancing healing of diabetic foot ulcer: a randomized double-blind controlled trial

**DOI:** 10.1007/s00403-023-02657-8

**Published:** 2023-07-22

**Authors:** Eliza Miranda, Kusmarinah Bramono, Em Yunir, Mirta H. Reksodiputro, Oki Suwarsa, Iris Rengganis, Alida R. Harahap, Decy Subekti, Suhendro Suwarto, Hayun Hayun, Saptawati Bardosono, Joko C. Baskoro

**Affiliations:** 1 Department of Dermatology and Venereology, Faculty of Medicine, Universitas Indonesia, Jakarta, Indonesia; 2 Department of Internal Medicine, Faculty of Medicine, Universitas Indonesia, Jakarta, Indonesia; 3 Department of Otorhinolaryngology, Faculty of Medicine, Universitas Indonesia, Jakarta, Indonesia; 4 Department of Dermatology and Venereology, Faculty of Medicine, Universitas Padjajaran, Sumedang, Indonesia; 5 Department of Clinical Pathology, Faculty of Medicine, Universitas Indonesia, Jakarta, Indonesia; 6 Faculty of Medicine, Oxford University Clinical Research Unit Indonesia, Universitas Indonesia, Jakarta, Indonesia; 7 Faculty of Pharmacy, Universitas Indonesia, Depok, Indonesia; 8 Department of Nutrition, Faculty of Medicine, Universitas Indonesia, Jakarta, Indonesia

**Keywords:** Colonization pattern, Diabetic foot ulcer, IL-1α, LL-37, TNF-α, Wound healing

## Abstract

Wound healing in DFU (diabetic foot ulcer) has prolonged inflammation phase and defective granulation tissue formation. LL-37 has antimicrobial property, induces angiogenesis, and keratinocyte migration and proliferation. This study analyzes the efficacy of LL-37 cream in enhancing wound healing rate and decreasing the levels of IL-1α, TNF-α, and the number of aerobic bacteria colonization in DFU with mild infection. This study was conducted from January 2020 to June 2021 in Jakarta. Subjects were instructed to apply either LL-37 cream or placebo cream twice a week for 4 weeks. Wounds were measured on days 7, 14, 21, and 28 and processed with ImageJ. The levels of LL-37, IL-1α, and TNF-α from wound fluid were measured using ELISA. The number of aerobic bacteria colonization was counted from the isolate grown in culture. The levels of LL-37 in DFU at baseline were equally low in both groups which were 1.07 (0.37–4.96) ng/mg protein in the LL-37 group and 1.11 (0.24–2.09) ng/mg protein in the placebo group. The increase in granulation index was consistently greater in the LL-37 group on days 7, 14, 21, and 28 (*p* = 0.031, 0.009, 0.006, and 0.037, respectively). The levels of IL-1α and TNF-α increased in both groups on days 14 and 21 (*p* > 0.05). The decrease in the number of aerobic bacteria colonization was greater in the LL-37 group on days 7, 14 and 21, but greater in the placebo group on day 28 (*p* > 0.05). In conclusion, LL-37 cream enhanced the healing rate of DFU with mild infection, but did not decrease the levels of IL-1α and TNF-α and the number of aerobic bacteria colonization. This trial is registered at ClinicalTrials.gov, number NCT04098562.

## Introduction

Diabetes is a chronic disease which affects 451 million people around the world [1]. One of the most common complications associated with diabetes is DFU (diabetic foot ulcer) which not only reduces quality of life, but also becomes a worldwide economic burden [2, 3]. As many as 40–70% of lower extremities amputations occurred in diabetic participants and 85% of them were preceded with DFU [4].

There are several factors that contribute to the formation of DFU, such as local inflammation, reduction of growth factors, uncontrolled proteolysis [5, 6], insufficient phagocytosis by macrophages, inadequate cell migration and angiogenesis [7], and increase of infection burden [8].

The majority of chronic ulcers respond well to conventional treatment. However, 15–20% of those ulcers fail to heal regardless of adequate treatment [9, 10]. Compared to the normal acute wound healing, chronic wounds often have prolonged inflammation phase and defective granulation tissue formation [11].

During the early stage of normal wound healing process, natural immune cells produce proinflammatory cytokines which are responsible for inducing the immunity system to secrete AMP (antimicrobial peptide), attract leukocytes, and create an environment protected from microbial infection. IL-1 and TNF-α are able to induce the expression of AMP [12]. AMPs are a mediator of body’s natural immune response. AMPs include β-defensins (hBDs), cathelicidin/LL-37, lysozyme, RNase 7, elafin, psoriasin, dermcidin, adrenomedullin, secretory leukocyte protease inhibitor, dan neutrophil gelatinase-associated lipocalin [13]*.* In human, the gene of cathelicidin encodes an 18 kDa inactive precursor protein called human cationic antimicrobial peptide-18 (hCAP18) which then releases terminal C amino acid chain and turns into active LL-37 peptide [14]. Other than its antimicrobial function, LL-37 has other biological effects such as neutralizing endotoxins, chemokine-mimicking activities, immunomodulation, cytokine production, histamine release, angiogenesis, and wound healing [9, 15–19].

Cathelicidin plays an important role in the pathogenesis of DFU due to its antimicrobial action and induction of angiogenesis and keratinocyte migration and proliferation. These activities are crucial in adequate wound healing. Immunohistochemistry staining of DFU biopsy revealed low and even zero concentration of LL-37 expression, although, in normal skin, there was optimal concentration of LL-37 [20]. There is currently no research on the LL-37 concentration of DFU fluid.

A randomized controlled trial on the efficacy and safety of treatment with LL-37 by Grönberg et al. [21] showed LL-37 was effective for the treatment of venous leg ulcer in 34 subjects. Significantly good response was achieved after 4 weeks of twice weekly application of 0.5 mg/mL and 1.6 mg/mL LL-37. Unfortunately, these results were not applicable to DFU due to different pathophysiology between DFU and venous leg ulcer. There is currently no clinical trial on the efficacy of cathelicidin for DFU in Indonesia or other countries.

LL-37 is a promising treatment for chronic wounds because of its antibiofilm property and because it is part of host cells which act simultaneously to eradicate causative bacteria. It interacts with keratinocytes and fibroblasts to induce wound closure [22, 23]. In addition, infective agents in chronic ulcers are commonly polymicrobial species; therefore, treatment with antibiotic for a specific bacteria will be ineffective [24]. Therefore, this study aims to analyze the efficacy of LL-37 cream in enhancing wound healing rate, decreasing the levels of IL-1α, TNF-α, and altering the pattern and number of aerobic bacteria colonization in DFU with mild infection.

## Materials and methods

### Overall study design

This study began with a laboratory experimental study on the validation and stability test of LL-37 in cream vehicle. It then proceeded with the randomized, double-blind controlled trial on the efficacy of LL-37 cream for DFU healing. The laboratory experimental study was conducted at Quality Testing Service Laboratory, Faculty of Pharmacy, Universitas Indonesia, Depok. The clinical trial was conducted at Outpatient Clinic of Internal Medicine Department, RSCM. Samples were analyzed at Eijkman Institute for Molecular Biology, while the levels of LL-37, IL-1α and TNF-α were measured at Integrated Laboratory Faculty of Medicine, Universitas Indonesia. Sample collection and data analysis for the clinical trial were conducted from January 2020 to June 2021. Randomization sequence was generated using a computer with a 1:1 allocation using random block sizes of 8. The LL-37 and placebo creams were prepacked in identical opaque jars and consecutively numbered for each participant. Determination of whether a participant would be treated with LL-37 cream (group A) or with placebo cream (group B) was prepared by a statistical supervisor with no clinical involvement in the trial and was unknown to any of the researchers. After a participant signed the consent form, they were assigned a prepacked jar on which the letter “A” or “B” was written. Both participants and researchers were kept blinded to the allocation of the treatment. This study was approved by the FKUI-RSCM Medical Research Ethical Committee in Jakarta and it complied with the Declaration of Helsinki guidelines. Written informed consent was obtained from every subject. They also signed informed consent regarding publishing their data and photographs. This trial is registered at ClinicalTrials.gov, number NCT04098562.

### Study population

The study population was DFU participants coming to Outpatient Clinic of Internal Medicine Department RSCM, Clinic of Dermatology and Venereology Department RSCM, and Clinic of Internal Medicine Department RSP (RSUP Persahabatan). The target population was DFU participants who met inclusion criteria: uninfected DFU or DFU with mild infection according to IDSA; aged 18–60 years old, ABI 0.9–1.3; wound area ≥ 2 cm^2^; wound no deeper than subcutaneous layer; and without systemic infection, osteomyelitis, septic arthritis, or fasciitis. The exclusion criteria: gangrene, oral corticosteroid consumption within 7 days prior to the start of study; oral and topical antibiotic use within 2 days prior to the start of study; and terminal kidney failure.

### Drug substance, drug product, and diluent

The validation test was conducted to prove that the HPLC (high-performance liquid chromatography) method could provide consistent measurement results of LL-37 levels in accordance with the established and well-documented specifications based on AOAC (Association of Official Analytical Chemists) and ICH (International Conference on Harmonization) criteria [25, 26]. The accelerated stability test was then conducted to see the stability of LL-37 level in cream for 12 weeks. The stability test consisted of physical stability and concentration of LL-37. Physical stability test was conducted by storing cream at temperatures of 40 ± 2 °C, 28 ± 2 °C, and 4 ± 2 °C for 12 weeks, then observing changes in color, odor, consistency, and pH every 2 weeks and measuring the concentration of LL-37 at 0, 1 and 3 months. The stability test of LL-37 cream concentration was conducted to determine the shelf life of cream to maintain a minimum concentration of 90% at various storage temperatures.

The LL-37 cream contained purified LL-37 substance from Isca Biochemicals, Exeter, UK. An LL-37 solution containing 50 mg of LL-37 and 20 g of purified water was added to 100 g of base cream to reach an LL-37 concentration of 0.5 mg/g. The cream formulation consisted of 7 g of stearic acid, 2 g of cetyl alcohol, 0.5 g of TEA (triethanolamine), 20 g of paraffinum liquidum, 10 g of glycerin, purified water (until the formulation reached 100 g), and 5% citric acid to reach a pH of 6.28 in room temperature. The creams were then distributed and packed in 20 jars, so each contained 5 g of LL-37 cream. The placebo cream was exactly 100 g of base cream without the LL-37 solution. It was also distributed and packed in 20 jars. The placebo cream was matched to the LL-37 cream for volume, color, consistency, and smell.

### Sample size

Kartika et al. [27] reported the means of granulation index delta percentage of DFU participants receiving both topical autologous advanced platelet-rich fibrin and hyaluronic acid and participants receiving 0.9% NaCl solution (control) were 41.7 (± 13.8)% and 24.6 (± 8.8)%, respectively. Using these numbers, the minimum sample size for detecting a difference between the means of two independent groups was 16. This yielded a statistical power of 84.02% with a significance level of 0.05. To compensate for participant dropout during the study, 10% was added so the final sample size was 18.

### Outcome measures

Subjects were asked to come twice a week, for 4 consecutive weeks. Every subject was given standard treatment for DFU, namely washing the wound with normal saline (0.9% NaCl) and removing necrotic tissue using sharp debridement method.

After wound debridement, measurement of wound area was conducted using planimetric method and digital photography with two rulers. The entire surface of the wound and rulers were photographed using a Canon PowerShot G7X digital camera (20.20 megapixels). The image was taken perpendicularly to form an angle of 90° to the wound surface at a distance of 30 cm, with good room lighting. The photos were then transferred to a computer. Photos were processed using ImageJ. At first, the wound size was calibrated based on the ruler scale on the edge of the wound. Then the edges of the wound were traced using mouse cursor for the calculation of the surface area. In addition to the wound edges, the amount of granulation tissue was also traced using mouse and the area was calculated. This was repeated several times at different times to see the progress of wound healing. The granulation index was calculated by dividing the granulation tissue area by the measured wound area.

Subsequently, the wound swab was taken to examine the number and pattern of aerobic bacteria colonization. Wound swabs were taken with Puritan Calcium Alginate Swab 25-806 1PA (Puritan, USA) using the Levine wound swab technique and a sterile square marker made of metal. The swab tip was immediately inserted into a tube containing 5 mL of phosphate-buffered saline (PBS) and immediately delivered to the Eijkman Microbiology Laboratory.

Wound fluid collection for the examination of LL-37, IL-1α, and TNF-α was performed using Whatman^™^ filter paper size 54 (GE Healthcare, Chicago, Illinois, USA) coated with 3 M^™^ Tegaderm^™^ non-adherent contact layer dressing sterile (3 M, Maplewood, Minnesota, USA), then the wound was covered with 3 M^™^ Tegaderm^™^ transparent film dressing for 60 min [28–30]. Whatman paper that had absorbed wound fluid was put into a sterile tube and stored in a cooler box. Protein extraction using the Bradford method from both types of samples was carried out at Eijkman Molecular Biology Laboratory for further examination. The levels of IL-1α and TNF-α were measured using wound fluid samples obtained on day 1, day 14, and day 21 using the sandwich ELISA technique with the R&D System kit, Minneapolis, Minnesota, USA. The level of LL-37 was measured only on day 1 using the sandwich ELISA technique with the Hycult Biotech kit, Uden, Netherlands.

### Interventions

The cream requirement for each participant in both groups was 0.025 mL/cm^2^. The cream was applied topically on wound surface using a 1-mL syringe and was spread evenly with a spatula. The wound was subsequently dressed with 3 M^™^ Tegaderm^™^ (Maplewood, Minnesota, the USA).

### Statistical analysis

Data processing was performed using SPSS 20 program and Stata 17 program. Statistical analysis was performed using intention-to-treat method. Nominal variable analysis was performed using Chi-square test or Fisher’s exact test. Analysis of unpaired numerical variables between the two groups was carried out using unpaired *t* test if the data distribution was normal or Mann–Whitney test if the data distribution was not normal. Bivariate analysis was performed using robust Poisson regression to determine the prevalence ratio using Stata version 17 program.

## Results

### Laboratory test

Before the clinical trial, a laboratory experimental test was conducted on the stability of LL-37 in cream vehicle. The validation of the stability test method for 0.5 mg/mL LL-37 cream was carried out using the HPLC method. The system suitability test, correlation coefficient, selectivity of analysis method, accuracy, and precision all met the selection criteria (Table 1). The accelerated stability test of LL-37 cream found that LL-37 cream melted at 40 °C, while remaining stable at 4 °C and 28 °C. The results of the physical stability test of LL-37 cream showed no change in pH, color, and odor at all temperatures. LL-37 cream could survive with an active ingredient concentration of > 90% after being made and could be stored at 4 °C for 99 months, at 28 °C for 75 months, and at 40 °C for 16 months.

**Table 1:** Baseline demographic and clinical characteristics

Characteristics	LL-37 (*n* = 13)	Placebo (*n* = 12)
Age (years), mean (SD)	53 (± 5)	52 (± 5)
Male/female	6/7	7/5
Education		
No education	0	1
Elementary school	3	1
Middle school	1	0
High school	7	7
Diploma/bachelor	1	3
Masters	1	0
Job		
Unemployed	6	5
Employee	5	3
Entrepreneur	2	4
Diabetes duration		
< 1 year	1	1
1–5 years	2	3
> 5–10 years	1	3
> 10 years	9	5
Antidiabetic drug		
Oral	8	7
Insulin	2	1
Combination	3	4
Anticoagulant	4	4
Neuropathy	10	6
DFU duration		
< 2 weeks	1	1
2–4 weeks	2	3
> 4 weeks	10	8
DFU location		
Digit	4	1
Forefoot	3	5
Midfoot	6	2
Hindfoot	0	3
Ankle	0	1
Infection severity		
Uninfected	9	8
Mild	4	4
Wound area using planimetry (cm^2^), median (min–max)	5.00 (2.10–50.25)	5.05 (2.10–18.36)
Blood glucose (mg/dL)		(*n*)
< 200	10	9
200–300	2	2
> 300	1	1
HbA1c (%)		
< 7	5	4
> 7	8	8
LL-37, median (min–max)		
(ng/mg protein)	1.07 (0.37–4.96)	1.11 (0.24–2.09)
(10^–3^ µM)	0.2 (0.1–1.1)	0.3 (0.1–0.5)

*DFU* Diabetic foot ulcer, *SD* standard deviation

### Participant flow and baseline data

**Fig. 1:**
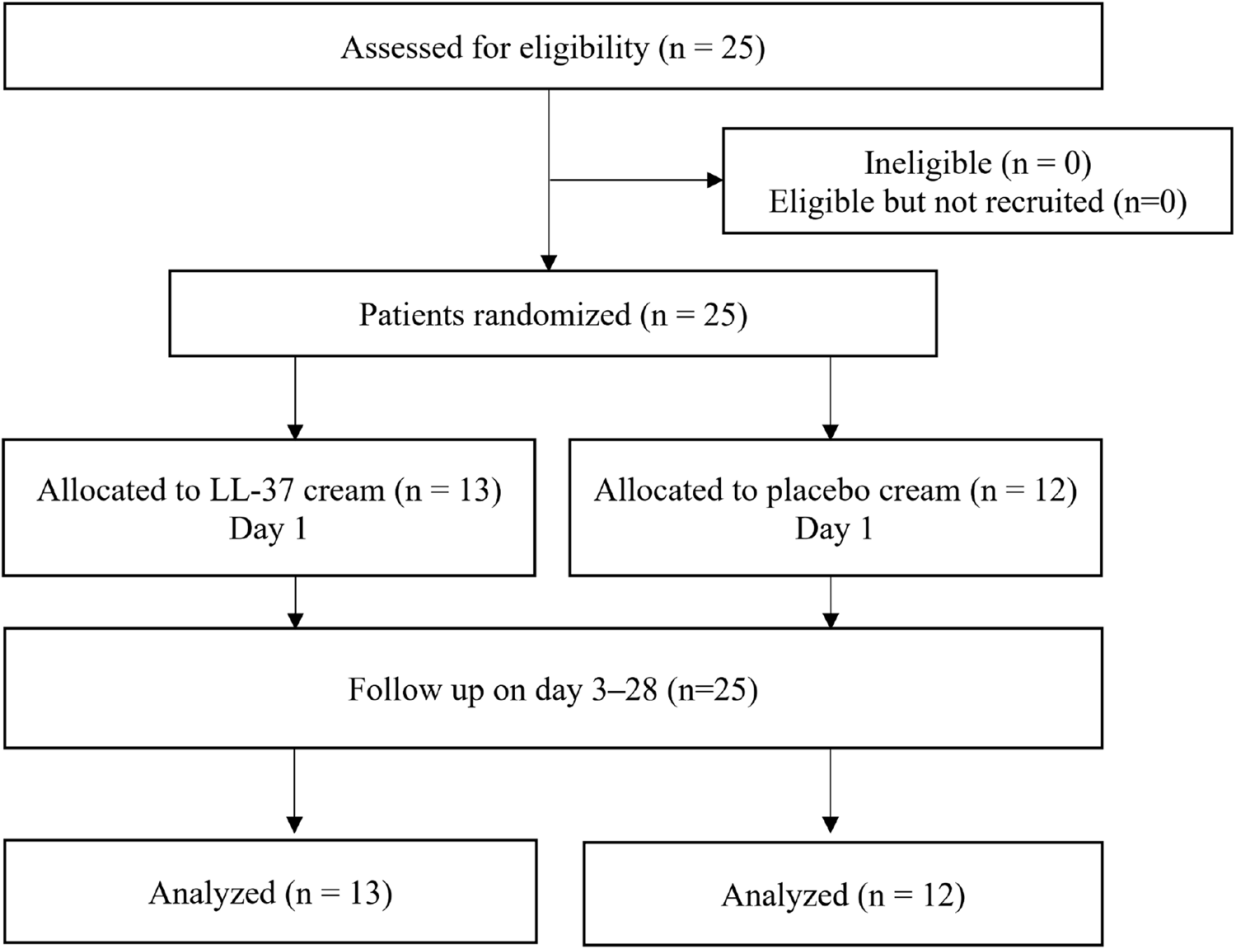
Flow diagram of the trial

Figure 1 shows the study flow chart. Twenty-five participants with DFU were screened between January 2020 and June 2021 in this randomized double-blind controlled trial. Twenty-three participants were screened from the Outpatient Clinic of Internal Medicine Department RSCM, one from the Outpatient Clinic of Dermatology and Venereology Department RSCM, and one from the Outpatient Clinic of Internal Medicine Department RSP. Out of 25 participants, 13 were randomized to receive LL-37 cream and 12 received placebo cream. Participants attended clinic visits twice weekly for 4 consecutive weeks to receive the allocated treatments, namely on days 1 (baseline), 3, 7, 10, 14, 17, 21, 24 and 28. During this follow-up period, no participant withdrew from this study, was excluded, or was lost to follow-up; thus, data from all 25 participants were available for the intention-to-treat analysis.

The baseline demographic and clinical characteristics are summarized in Table 1. Most subjects had diabetes for longer than 10 years, consumed only oral antidiabetic, did not consume oral anticoagulant, and had neuropathy. Most subjects had DFU for longer than 4 weeks. The most common DFU location in the LL-37 group was midfoot and in the placebo group was forefoot. The DFUs were mostly uninfected. The mean wound area measured using planimetry at baseline was similar in both groups. The median LL-37 level in wound fluid at baseline was 1.07 (0.37–4.96) ng/mg protein in the LL-37 group and 1.11 (0.24–2.09) ng/mg protein in the placebo group (*p* = 0.44), so there was no difference in the distribution of LL-37 levels in both intervention groups.

### Efficacy of LL-37 on wound healing

**Fig. 2:**
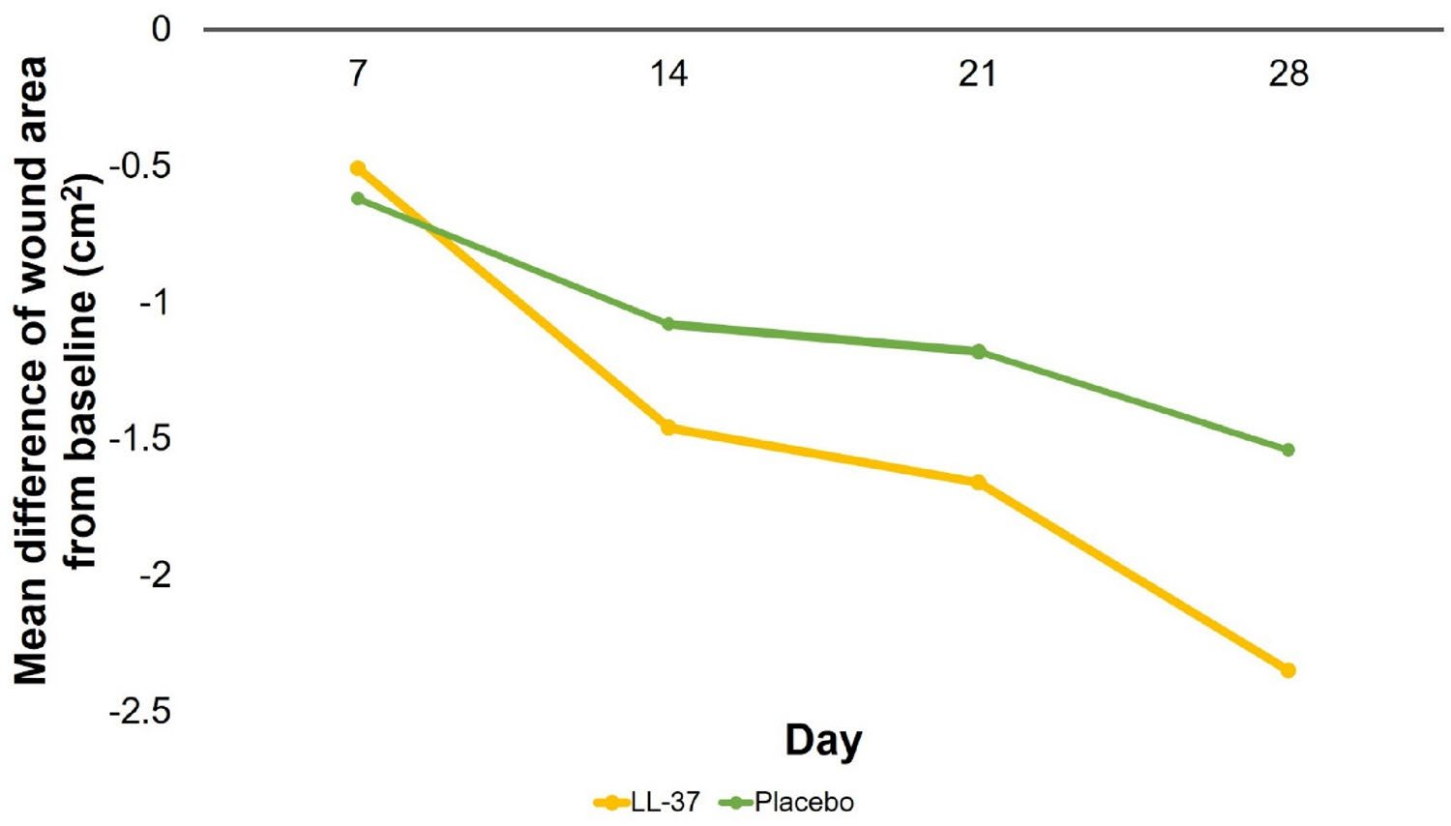
Mean difference of wound area from baseline using ImageJ

The decrease in wound area (Δ wound area) compared to day 1 was greater in the LL-37 group than the placebo group on days 14, 21, and 28 (Fig. 2). The greatest difference of Δ wound area between two groups was found on day 28, which was − 2.35 (± 3.86) cm^2^ in the LL-37 group and − 1.54 (± 0.94) cm^2^ in the placebo group, although, on each day, the differences were not significant based on independent *t* test (*p* > 0.05).

The granulation index in the LL-37 group reached 100% (1.00) on day 14 whereas in the placebo group on day 28. There was a consistently and significantly greater increase in granulation index (*p* < 0.05) in the LL-37 group compared to the placebo group on each day (Table 2). The most significant difference occurred between day 21 compared to day 1 (*p* = 0.006). The mean delta granulation index in the LL-37 group was 0.64 (± 0.30) while in the placebo group was 0.30 (± 0.26).

**Table 2:** Mean difference of granulation index from baseline

Day	LL-37 (*n* = 13) Mean (SD)	Placebo (*n* = 12) Mean (SD)	*p* value
7	0.41 (± 0.26)	0.16 (± 0.28)	0.031*
14	0.60 (± 0.31)	0.27 (± 0.26)	0.009*
21	0.64 (± 0.30)	0.30 (± 0.26)	0.006*
28	0.64 (± 0.31)	0.36 (± 0.31)	0.037*

Independent *t* test

*SD* Standard deviation

*Significant difference (*p* < 0.05)

Based on the ROC curve, the cutoff point of the granulation index delta between the LL-37 and placebo groups was ≥ 0.41 with sensitivity of 81.8%, specificity of 66.7%, AUC (area under the curve) of 75.8%, and 95% CI of 54.2–97.3. As many as 11 of 13 subjects in the LL-37 group showed an increase in granulation index higher than 0.41 on day 21 compared to only 3 of 12 subjects in the placebo group. This difference was significant (*p* < 0.05).

IL-1α level in the LL-37 group on day 1 was much higher with a median of 38.86 (7.94–124.56) pg/mg compared to the placebo group with a median of 12.07 (1.97–222.34) pg/mg and the difference was significant based on Mann–Whitney test (*p* = 0.04). On day 21, there was a greater increase in IL-1α level compared to day 1 in the LL-37 group with a median of 137.78 (− 84.17–11,040.61)% than in the placebo group with a median of 132.72 (− 90.95–7431.35)%, although it was not significant (*p* > 0.05) (Table 3).

**Table 3:** Median of inflammation marker levels

Days	IL-1α (min–max)		*p* value	TNF-α (min–max)		*p* value
	LL-37	Placebo		LL-37	Placebo	
1	38.86 (7.94–124.56) (*n* = 13)	12.07 (1.97–222.34) (*n* = 12)	0.04*	7.97 (2.88–34.19) (*n* = 13)	5.11 (2.65–12.76) (*n* = 12)	0.23
14	52.03 (6.00–191.35) (*n* = 13)	37.59 (3.90–590.00) (*n* = 12)	0.91	7.23 (1.37–370.53) (*n* = 13)	6.91 (1.72–278.13) (*n* = 12)	0.51
21	46.51 (6.76–1175.00) (*n* = 11)	33.70 (3.80–216.41) (*n* = 9)	0.27	11.16 (3.05–2580.00) (*n* = 11)	11.38 (2.14–156.88) (*n* = 9)	0.73
%Δ 1–14	32.11 (− 85.94–1714.30) (*n* = 13)	138.53 (− 90.31–4943.86) (*n* = 12)	0.09	13.59 (− 96.00–2047.01) (*n* = 13)	70.61 (− 72.23–3631.32) (*n* = 12)	0.19
%Δ 1–21	137.78 (− 84.17–11,040.61) (*n* = 11)	132.72 (− 90.95–7431.35) (*n* = 9)	1.00	52.38 (− 67.37–25,033.22) (*n* = 11)	67.01 (− 45.00–5822.94) (*n* = 9)	1.00

Mann–Whitney test

*Significant difference (*p* < 0.05); %Δ1-14: difference percentage between day 14 and baseline; %Δ1-21: difference percentage between day 21 and baseline

TNF-α level in the LL-37 group on day 1 was higher with a median of 7.97 (2.88–34.19) pg/mg compared to the placebo group with a median of 5.11 (2.65–12.76) pg/mg and the difference was not significant based on Mann–Whitney test (*p* = 0.23). On day 21, there was a greater increase in TNF-α level compared to the 1st day in the placebo group with a median of 67.01 (− 45.00–5822.94)% than in the LL-37 group with a median of 52.38 (− 67.37–25,033.22)%, although it was not significant (*p* > 0.05) (Table 3).

On day 21, bivariate analysis was performed on 20 subjects because there were 5 subjects whose wounds had closed so that the levels of IL-1α and TNF-α could no longer be measured. Using the cutoff point of the granulation index on the ROC curve, the variables in the bivariate analysis that caused a significant increase in the granulation index were the percentage of delta IL-1α and TNF-α between day 21 and day 1 (Table 4).

**Table 4:** Association between inflammation marker difference on day 21 compared to baseline and excellent granulation index growth (*n* = 20)

Inflammation marker	PR (CI 95%)	*p* value
IL-1α	1.000069 (1.000018–1.00012)	0.008*
TNF-α	1.000031 (1.000007–1.000054)	0.013*

Robust Poisson regression; *PR* *prevalence ratio*

*Significant difference (*p* < 0.05)

**Fig. 3:**
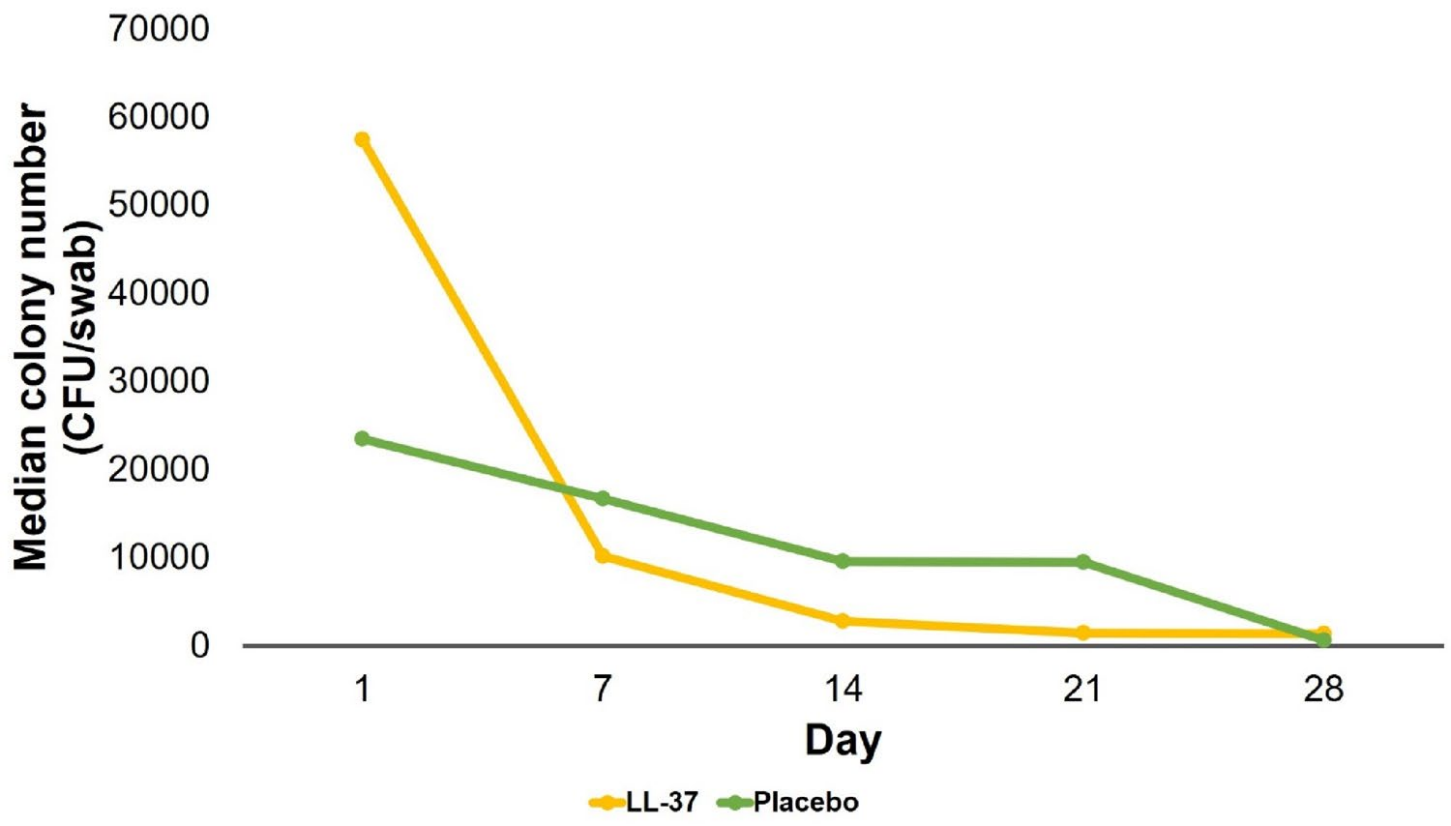
Median colony number

The frequency of aerobic bacteria species at baseline was dominated by *S. aureus*. As many as 10 of 27 bacteria (37.1%) isolated in the LL-37 group and 9 of 20 bacteria (45%) isolated in the placebo group were *S. aureus*. The second most common aerobic bacteria species was *Pseudomonas sp.* As many as 7 of 27 bacteria (25.9%) in the LL-37 group and 2 of 20 bacteria (10%) in the placebo group were *Pseudomonas sp.* On day 28, there was a decreasing trend of the two most common bacteria isolated from DFU (*S. aureus* and *P. aeruginosa/Pseudomonas sp.*) which were equivalent between the two groups. The distribution of Gram-positive bacteria in the LL-37 group was equivalent to the placebo group. Both on day 1 and day 28, there were more Gram-positive than Gram-negative bacteria in both groups. The decrease in the colony number on day 28 compared to day 1 in the placebo group was greater than in the LL-37 group (Fig. 3). The number decreased by − 10,412.50 (– 21,727,250–299,875) CFU/swab in the placebo group and – 1600 (– 4,953,225–252,475) CFU/swab in the LL-37 group, although it was not significant (*p* > 0.05).

During the trial, there was no side effect directly caused by the application of LL-37 cream. One of thirteen participants in the LL-37 group had mild irritant contact dermatitis due to excessive wound exudate and resolved after adding 3 M™ Tegaderm silicone foam dressing to absorb the exudate.

## Discussion

This study aimed to evaluate the efficacy of LL-37 cream in the treatment of DFU. At baseline, the median LL-37 levels in both groups was equally low with 1.07 (0.37–4.96) ng/mg protein in the LL-37 group and 1.11 (0.24–2.09) ng/mg protein in the placebo group. The low level of LL-37 in DFU obtained in this study is in accordance with Rivas-Santiago et al. [20] who reported that LL-37 expression very low and could not even be found in immunohistochemical examination of DFU biopsy tissue, whereas healthy skin showed optimal LL-37 levels. Data show that at least 0.2 M LL-37 is required for wound healing, 0.001 M for antibiofilm effect, 0.002 M for inhibition of neutrophil apoptosis, 0.011 M for angiogenesis, and 0.02 M for antimicrobial activity [14].

The primary efficacy parameter in this study is the granulation index. This is supported by a study by Iizaka et al. [31] showing that %WRI (wound red index), which is the percentage of the wound surface covered with healthy red granulation tissue, has an adequate predictive validity against wound closure and improvement in deep pressure ulcers. In this trial, the increase in granulation index was consistently and significantly higher in LL-37 group compared to placebo group on all days. In addition, the LL-37 group achieved a mean granulation index of 1.00 on day 14, in contrast to the placebo group on day 28. This indicates that LL-37 accelerates wound healing in DFU. In accordance with the result of this trial, Cao et al. [32] reported that wound healing in experimental rats experienced faster re-epithelialization and granulation tissue formation after receiving therapy with cathelicidin-OA1, which is a human cathelicidin analog obtained from a frog species. The mechanism of wound healing was through increased recruitment of macrophages to the wound site, proliferation of keratinocytes, and migration of fibroblasts. Carretero et al. [33] also supported the action mechanism of LL-37, reporting that 100 ng/mL LL-37 and 500 ng/mL LL-37 induced migration of keratinocytes.

Moreover, the proportion of study participants achieving granulation index increase higher than the cutoff of 0.41 on day 21 was significantly higher in the LL-37 group. This further supported the advantage of LL-37 in wound healing. This observation is likely related to evidence showing the mechanism of LL-37 on extracellular matrix and angiogenesis. A study showed that cathelicidin-NV could significantly increase the expression of MCP-1, TNF-α, TGF-β1, and VEGF. TGF-β1 plays an important role in the formation of extracellular matrix, while VEGF and MCP-1 increase migration and proliferation of endothelial cells to stimulate angiogenesis [34].

The mean wound area reduction in this trial is only slightly higher in the LL-37 group on days 14, 21 and 28 with no significant difference to the placebo group, not consistent with the granulation index increase. This is in contrast to a similar trial by Grönberg et al. [21] and another study by Wu et al. [34] who reported that LL-37 was able to reduce wound area and increase percentage of wound closure, respectively. This is likely due to the mechanism of LL-37 being more dominant in the early proliferation phase. More studies are needed to confirm this.

In this study, there was an increase in both IL-1α and TNF-α levels in both groups. However, the increase in inflammation markers in the LL-37 group is in accordance with the higher increase in granulation index, indicating that these markers, in fact, have roles in wound healing, contradictory to older theories saying that higher levels of IL-1α and TNF-α are harmful to the wound healing process. This is supported by a study by Werner et al. [35] who found that IL-1α and IL-1β produced by keratinocytes could stimulate fibroblasts in the dermis to produce KGF and GM-CSF which could trigger the proliferation and differentiation of keratinocytes. Another study by Salven et al. [36] reported that IL-1α injected subcutaneously in mice could stimulate a large angiogenesis response accompanied by infiltration of VEGF-expressing inflammatory cells.

Similar to IL-1α, the increasing TNF-α level in this study is beneficial in wound healing. Cao et al. [32] found that in the early stages of wound healing, cathelicidin-OA1 in the human monocyte-like cell line (THP-1) increased TNF-α which summoned macrophages to the wound site to accelerate wound healing through the secretion of TGF-β1. Moreover, a study by Frank et al. [37] in mice reported that wounds treated with TNF-α reached significantly faster epithelialization and neovascularization than wounds treated with TNF-α antibody or controls.

The high levels of inflammation marker in this study were probably because it was measured on days 14 and 21 during proliferation phase of wound healing, since there was a simultaneous increase in granulation index. This agrees with a study by Hübner et al. [38] in mice showing that the highest levels of IL-1α and TNF-α occurred 15–72 h after wound onset and decreased to basal levels after the proliferative phase was completed. The proliferative phase begins 3–10 days after the wound is formed and lasts up to 21 days [39].

**Fig. 4:**
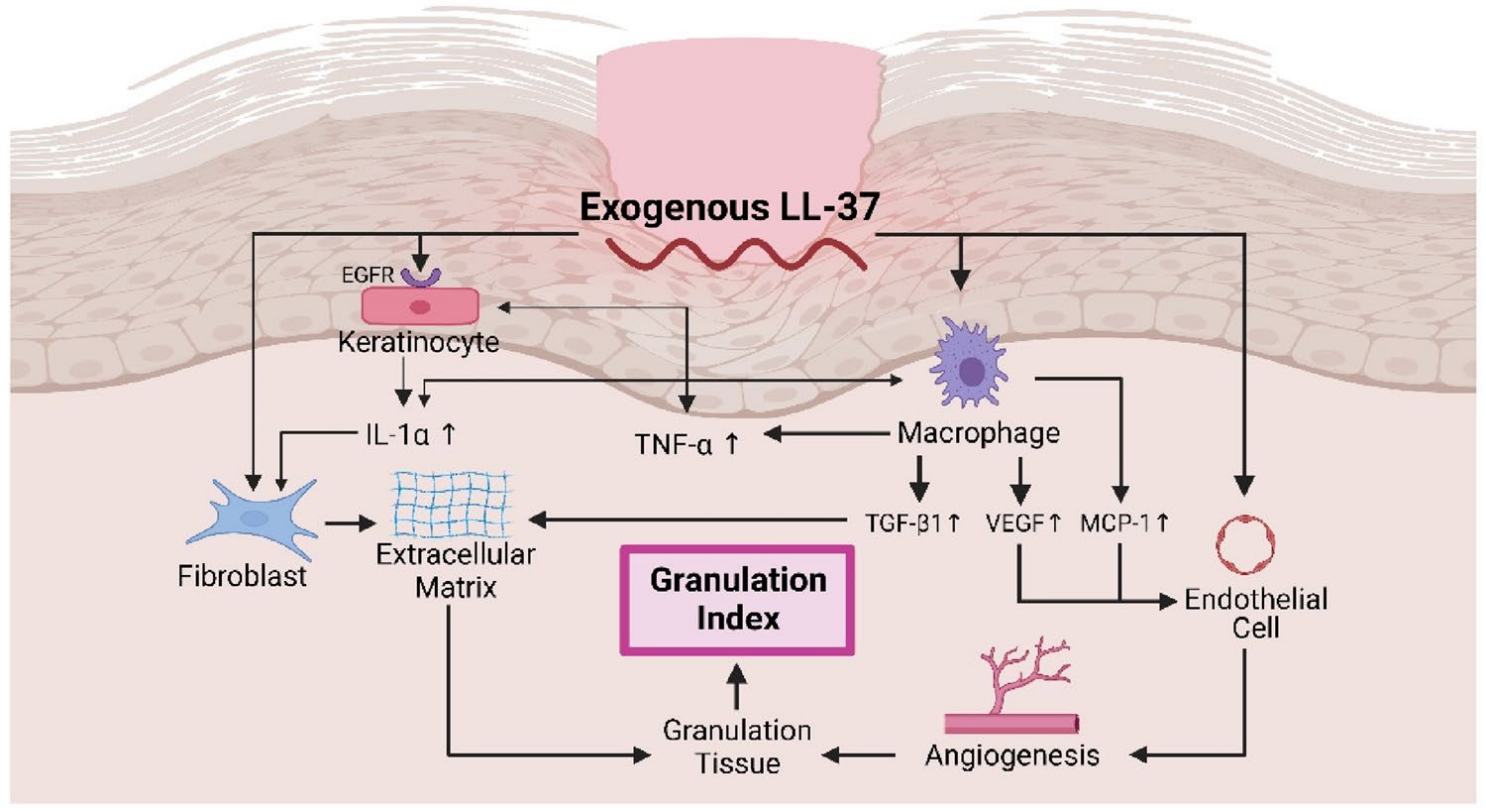
Proposed mechanism

Taken together, LL-37 stimulates macrophage to produce TGF-β1 which synthesizes extracellular matrix as well as VEGF and MCP-1 which enhance the migration and proliferation of endothelial cells to accelerate angiogenesis. In addition, LL-37 stimulates fibroblast to produce extracellular matrix. The growth of granulation tissue may result from the increase in IL-1α and TNF-α secreted by keratinocyte (through the activation of EGFR) and macrophage. IL-1α activates fibroblast to synthesize extracellular matrix and stimulate macrophage to secrete VEGF through endothelial cells for angiogenesis. TNF-α stimulates the proliferation and migration of keratinocyte. Eventually angiogenesis and the synthesis of extracellular matrix induce the growth of granulation tissue, and therefore granulation index (Fig. 4).

In this study, LL-37 was not able to significantly reduce the number of bacterial colonies. This is in contrast to the previous studies reporting the antimicrobial property of LL-37. It is likely due to mechanisms of resistance to LL-37 shown by some bacteria which have been reported in previous studies. These bacteria showing resistance to LL-37 are also the most frequently isolated bacteria in this study, namely *S. aureus, Pseudomonas sp.*, and *S. epidermidis*. Cao et al. [32] reported that cathelicidin-OA1 did not show direct antimicrobial activity on gram-positive bacteria such as *S. epidermidis*, *S. haemolyticus*, and *E. faecalis* or Gram-negative bacteria such as *P. aeruginosa*, despite using the highest concentration of 1 mM. Some bacteria such as *S. aureus* and *P. aeruginosa* can avoid the antimicrobial activity of LL-37 through the formation of biofilms. Luo et al. [40] reported that LL-37 could prevent the formation of biofilms by *S. aureus*, but could not damage the biofilms that had already been formed. Biofilms are able to increase antibiotic resistance in bacteria, thereby interfering with antimicrobial action and increasing infection. *P. aeruginosa* can also produce proteinases that can degrade LL-37 [41]. Therefore, the inability of LL-37 to reduce the colony number is probably because the dominating bacteria are already resistant. Moreover, there are some confounding factors which may influence microbiota in diabetic foot, such as hygiene, demographic characteristics, geographic origin of the patient, wound severity, blood glucose, and previous antibiotic treatment [42].

The main limitation of this study is the small study sample. Due to the large-scale social restriction policy implemented by the government during COVID-19 pandemic, the number of outpatients visiting RSCM and RSP diminished significantly, hindering participant recruitment. Another limitation was that the median IL-1α level was significantly higher in the LL-37 group at baseline. However, this study analyzed the difference percentage in both groups to prevent bias.

The trial was implemented for both sexes and there were equal distributions of education level, jobs, diabetes duration, ulcer duration, ulcer size, and HbA1c. Therefore, LL-37 cream can be reliably effective in all patients with DFU. However, it will be important to test the efficacy with more severely infected and larger DFU, as well as ulcers which are located in the hindfoot, ankle, and hallux. In conclusion, LL-37 cream effectively and safely accelerates wound healing in DFU.

## Data Availability

Raw data are not publicly available to preserve individuals’ privacy.
